# Presence of voids after three obturation techniques in band‐shaped isthmuses: a micro‐computed tomography study

**DOI:** 10.1186/s12903-021-01584-2

**Published:** 2021-05-01

**Authors:** Pengfei Zhang, Keyong Yuan, Qiaoqiao Jin, Fen Zhao, Zhengwei Huang

**Affiliations:** 1 Department of Endodontics, Shanghai Ninth People’s Hospital, Shanghai Jiao Tong University School of Medicine, College of Stomatology, Shanghai Jiao Tong University, Shanghai, 200011 China; 2 National Clinical Research Center for Oral Diseases, National Center for Stomatology; Shanghai Key Laboratory of Stomatology, Shanghai, 200011 China

**Keywords:** Isthmus, micro-CT, Obturation techniques, Voids

## Abstract

**Background:**

The objective of the present micro-computed tomography (micro-CT) study was to assess the presence of voids in band-shaped isthmuses obturated using three different filling techniques.

**Methods:**

Twenty-four artificial molar teeth with a band-shaped isthmus were allocated to three groups (n = 8) for obturation, according to the filling technique: single-cone (SC), continuous wave of condensation (CWC) or lateral condensation (LC). Obturation was performed with gutta-percha (GP) cones and iRoot SP (Innovative Bioceramix, Vancouver, Canada). Post-filling micro-CT scanning was performed. The percentage of filling materials and void volumes were calculated in the isthmus areas and data were analyzed using one-way ANOVA and Tukey tests.

**Results:**

The mean percentage of void volumes and corresponding filling percentages in the isthmus areas after obturation in the SC groups was 22.98 % ± 1.19 %, 77.02 % ± 1.19 %; in the CWC groups 10.46 % ± 2.28 %, 89.54 % ± 2.28 %; and in the LC groups was 13.14 % ± 1.85 %, 86.86 % ± 1.85 %, respectively.

**Conclusions:**

In band-shaped isthmus area, the obturation quality of CWC was superior to SC and LC techniques.

## Background

The goal of endodontic treatment is the removal of harmful microorganisms from the root canal to prevent or treat apical periodontitis [1]. Apart from the proper cleaning and shaping of the root canal, adequate obturation of the root canal system is also required after biomechanical procedures, to ensure the long-term success of endodontic treatment [2]. However, it is difficult to achieve this goal because of anatomical complexities of the root canal system and limitations in current preparation and filling techniques [3, 4]. Approximately 60 % of endodontic failures are due to insufficient obturation of the root canal system [3]. Inadequate obturation has been reported to result in voids within the canal filling and in the interface between filling and dentin, which permits bacterial movement in the coronal-apical direction or vice versa, potentially leading to reinfection or persistent apical periodontitis [5, 6]. Therefore, the association of high quality root canal obturation with treatment success has been emphasized [3, 5–8].

The variations and complexity of root canal anatomy such as isthmuses bring a key challenge in endodontic therapy of posterior teeth [4, 9]. An isthmus (also termed as a lateral interconnection, corridor, or transverse anastomosis) is a narrow, ribbon-shaped communication between two root canals [4, 9, 10]. A band-shaped isthmus is defined as a band-shaped corridor between root canals starting from the level where two canals merge (isthmus roof) and extending to the level where two root canals redivide (isthmus floor) [10]. The highest frequencies of isthmuses in human permanent teeth are found in the lower first molars, and their reported prevalence ranges from 50 to 80 % at the apical 5 mm level, evaluated using surgery and micro-CT methods [9–11].

The isthmuses remain one of the most difficult clinical challenges during root canal treatment, owing to accumulation of hard and/or soft tissue debris which can contain biofilms and intracanal microorganisms, after canal preparation within these areas [12, 13], leading to treatment failure. Therefore, the choice of root canal filling materials and obturation technique is very important in order to achieve a high quality seal in isthmus areas.

Most root canal filling techniques employ a core material, which is most commonly gutta-percha, and a sealer used to seal the space between gutta-percha (GP) and the canal wall. The traditional technique of lateral condensation (LC), is the most classic method of clinical root canal obturation and has been used for many years in numerous studies. Meanwhile, thermoplastic sealing techniques, such as continuous wave of condensation (CWC), have been reported to be advantageous for the management of irregular root canals [14, 15]. In addition, the single cone (SC) technique has become increasingly popular due to its easier implementation, better adaptation in three-dimensional preparation, low cost and short operation time [7, 14–17]when applied with a bioceramic sealer. Although a few studies [14–18] have used human molar teeth to evaluate the quality of obturation with different filling techniques, the variation was large because of complex canal anatomy shapes among different samples. For this reason, when measuring the percentage of voids in root canal fillings, three-dimensional (3D)-printed artificial teeth can provide more reliable information. However, there are still few reports involving a band-shaped isthmus [18].

Micro-computed tomography (micro-CT) scanning is an emerging technology with multiple applications in endodontic research [7, 9, 10, 14–18]. In terms of root canal filling, compared to conventional imaging techniques, such as scanning electron microscopy or confocal microscopy, micro-CT imaging is a nondestructive and visual method to calculate the volume of filling material without damaging the specimen, providing high-resolution 3D assessment of the quality of filling [15–21].

This study aimed to evaluate and compare the canal filling quality when performing different techniques on 3D-printed artificial teeth with band-shaped isthmuses by calculating the percentage of voids which was analyzed using micro-CT. The null hypothesis was that the percentage of voids in isthmus areas would not be influenced by filling technique.

## Methods

### Preparing 3D-printed artificial molar teeth

The teeth were customized samples reproducing the shape of the human mandibular first molar, which possessed Vertucci classification [4] type VI canals in the mesial root. Mesial-buccal (MB) and mesial-lingual (ML) canals with a 1 mm diameter and 4 mm distance at the orifice, were relatively straight and were joined together 5 mm from the apex and continued to the apex eventually. Thus, there was a root canal anatomy of “band-shape isthmuses” [10, 18] present in the mesial root of these teeth, with a roof and floor which exhibited a long, oval cross-sectional shape. The isthmus roof was at apex 5mm,and its floor was at apex 1mm.The buccal-lingual width of it was 1.5mm,and the mesial-distal width of it was 0.2mm. This was the focus of our research, so the follow-up processing only involved the mesial root, especially the apical 5 mm region. The image data with DICOM style acquired from the micro-CT (Skyscan 1172; Bruker-Micro-CT, Kontich, Belgium) scans was exported to Mimics® software (version 21.0; Materialise Corporation, Leuven, Belgium) to conduct 3D reconstruction and generate 3D printing model. Then 3D printing model data was exported to 3D printing software, through which the 3D-printed artificial molar teeth were produced at a printing precision of 0.016 mm on a 3D Systems Projet MJP3510 HD printer (3D Systems, Inc., Rock Hill, USA) with multi-jet printing technology (Multi-Jet Printing System, MJP).The crown of the teeth was generated by VeroWhite Plus RGD835 and the root was made of VeroClear RGD810 (Typrius,Inc.Beijing, China),which both had similar elastic modulus with respective part of human teeth and had a melting point at 375 and 350 °C respectively.

### Samples selection and root canal preparation

Based on the data of previous studies, a power beta of 0.95 by using G*Power 3.1 (Heinrich Heine University, Düsseldorf, Germany) was set, indicated a minimum expected sample size for each group should be six requiring to observe the same effect(3.48) [15, 19]. Then, eight 3D-printed artificial molar teeth for each obturation group were selected for this study to assess the void percentage in isthmus areas. The crowns of the artificial teeth were cut horizontally from the cervix for the convenience of operation. Then working length (WL) was determined using an ISO size #10 K-file (Dentsply, Switzerland) inserted into the canals until the tip was just visible beyond the apex with a dental operating microscope (Zeiss, Gottingen, Germany) at a magnification of 8× and was measured by deducting 1 mm from this length. Then, both mesial canals were instrumented, applying a crown-down technique using ProTaper Gold Ni-Ti system (Dentsply, Switzerland) coupled with the Dentsply X-Smart motor (Dentsply, Switzerland) in the sequence recommended by the manufacturer, reaching to F3. A new file was used on each specimen. Every time the instrument was withdrawn, the canals were irrigated with physiological saline delivered by a 30-gauge needle (Monoject, Covidien, Dublin, Ireland). After preparation, the canals were dried with paper points (Dentsply, Switzerland).

After preparation, to evaluate the consistency of canal shapes and homogeneity of isthmus figures, three teeth were randomly selected for micro-CT scanning, and the obtained images were overlapped [21].

### Root canal filling

The overall 24 specimens were randomly allocated into three groups containing eight teeth each group. These specimens were filled using the single-cone (SC), lateral compaction (LC), or continuous wave of condensation (CWC) technique. The iRoot SP (Innovative Bioceramix, Vancouver, Canada) was used as the root canal sealer according to the manufacturers’ instructions in all three obturation procedures.

Group 1 was obturated using the SC technique [14–18]. The canals were first fully injected with iRoot SP via a 24-gauge needle tip provided by the manufacturer. The tip was slowly pulled toward the orifice from the point of engagement in the canal. Subsequently, a lentulo spiral was placed in the canal, to allow a thin sealer to cover the canal wall evenly. Then, a single F3 ProTaper GP cone (Dentsply, Switzerland) modified to the working length with sealer was slowly inserted into the canals. A heat plugger (B&L Biotech, Ansan, Republic of Korea)was used to cut excess filling material at the orifice level, and the cone was vertically condensed using a cold plugger.

Group 2 was obturated using the CWC technique [15, 18, 19]. The root canals were coated with a thin layer of sealer using a paper point, then the F3 GP cone (Dentsply, Switzerland) was fitted as a master cone with sealer placed to the WL. A B&L-alpha II tip (B&L Biotech, Ansan, Republic of Korea) setted at 180 °C was inserted into the canal to cut the master cone, retaining only the apical GP (5 mm). Next, the coronal portion of the canal was backfilled with B&L-Beta (B&L Biotech, Ansan, Republic of Korea) setted at 200 °C to the canal orifice, and vertically condensed with suitable hand pluggers.

Group 3 was obturated using the LC technique [7, 14]. The appropriate 35/0.02 tapered GP cone (Dentsply, Switzerland) modified to the WL as a master cone, was coated with a thin layer of sealer and slowly inserted into the root canal. Then the lateral compaction procedure was performed with a size 25 nickel-titanium finger spreader (Dentsply Tulsa, Switzerland) and size 20/0.02 taper accessory GP cones (Dentsply, Switzerland). After that, the coronal excess was seared off at the canal entrance and the remaining filling material was vertically condensed using a cold plugger.

A single professional endodontist(Z.W.H) with more than 15 years’ clinical experience performed all root canal preparation and filling procedures to ensure consistency. After obturation, specimens were stored at 37 °C with 100 % humidity for 2 weeks to confirm that the filling materials had set completely.

### Computerized microtomography and microscopy observation

Because the band-shaped isthmuses were in the apical 5 mm region of the specimen, first, three artificial tooth specimens after preparation were randomly selected to assess the prepared canal space shapes and discrepancies of isthmus figures among specimens by using the overlapped micro-CT images.

The obturated mesial roots of 3D-printed artificial teeth were scanned using high-resolution micro-CT as previously reported [21], operated at 100 kV and 278 mA using a 0.5 mm-thick aluminum filter and 40 % beam-hardening reduction, which had rotational steps of 0.5° and a cross-sectional pixel size of 18.30 mm. Scanning was conducted at 360° rotations around the vertical axis with a camera exposure time of 500 ms. The detector was calibrated before each scan, to minimize ring artifacts. From these scans, cross-sectional images were acquired using NRecon software (version 1.6.9.18; Bruker-Micro-CT).Then, the image data with DICOM style was exported to Mimics® software to conduct 3D reconstruction. The range of measurements was 5mm from the mesial root apex. The grayscale range was determined in a density histogram by using a global threshold method. With regard to filling material segmentation (GP and sealer), threshold values ranged from 40 to 255 Vm, and a grayscale range between 0 and 40 Vm was designated as a void. Finally, 3D models of isthmus region at 5mm from the mesial root apex were created for volumetric calculations using Mimics® software. Select the filling material pattern in the 3D model, and then click “Threshold Region Grow” to acquire filling material volume (Filling Vol). Then, The volume of voids was calculated by subtracting the Filling Vol from the post-preparation isthmus volume (Isthmus Vol). The percentage of voids (Voids Vol %) of the isthmus was calculated as follows:

$${\mathrm{Voids\,Vol}}\,\% = \left( {{\mathrm{Isthmus\,Vol}} - {\mathrm{Filling\,Vol}}} \right) \times 100/{\mathrm{Isthmus\,Vol}}$$

Because the point of this study focused on the band-shape isthmus present in the apical 5 mm region of the mesial root, in each specimen, the volume of the filling and voids in the isthmus were measured, and the percentage of voids and filling were calculated.

After micro-CT analysis, the roots were horizontally crossed-sectioned precisely at the middle of the isthmus, which was 3 mm away from the apex, using a low speed diamond wheel (Isomet, Buelhler Ltd, Dusseldorf, Germany) under water cooling. Each cross-sectioned surface was observed under a microscope at 16× magnification.

### Statistical analysis

The Shapiro-Wilk test was used to verify whether the data were normally distributed. The data showed normal distribution and homogeneous variance as well as the mean percentage. One-way ANOVA was used to assess differences in the void percentages among the three groups. GraphPad Prism 8 software (GraphPad Software Inc, La Jolla, CA, USA) was used for all analyses. A *P* value of < 0.05 was considered to indicate statistical significance.

## Results

**Fig. 1:**
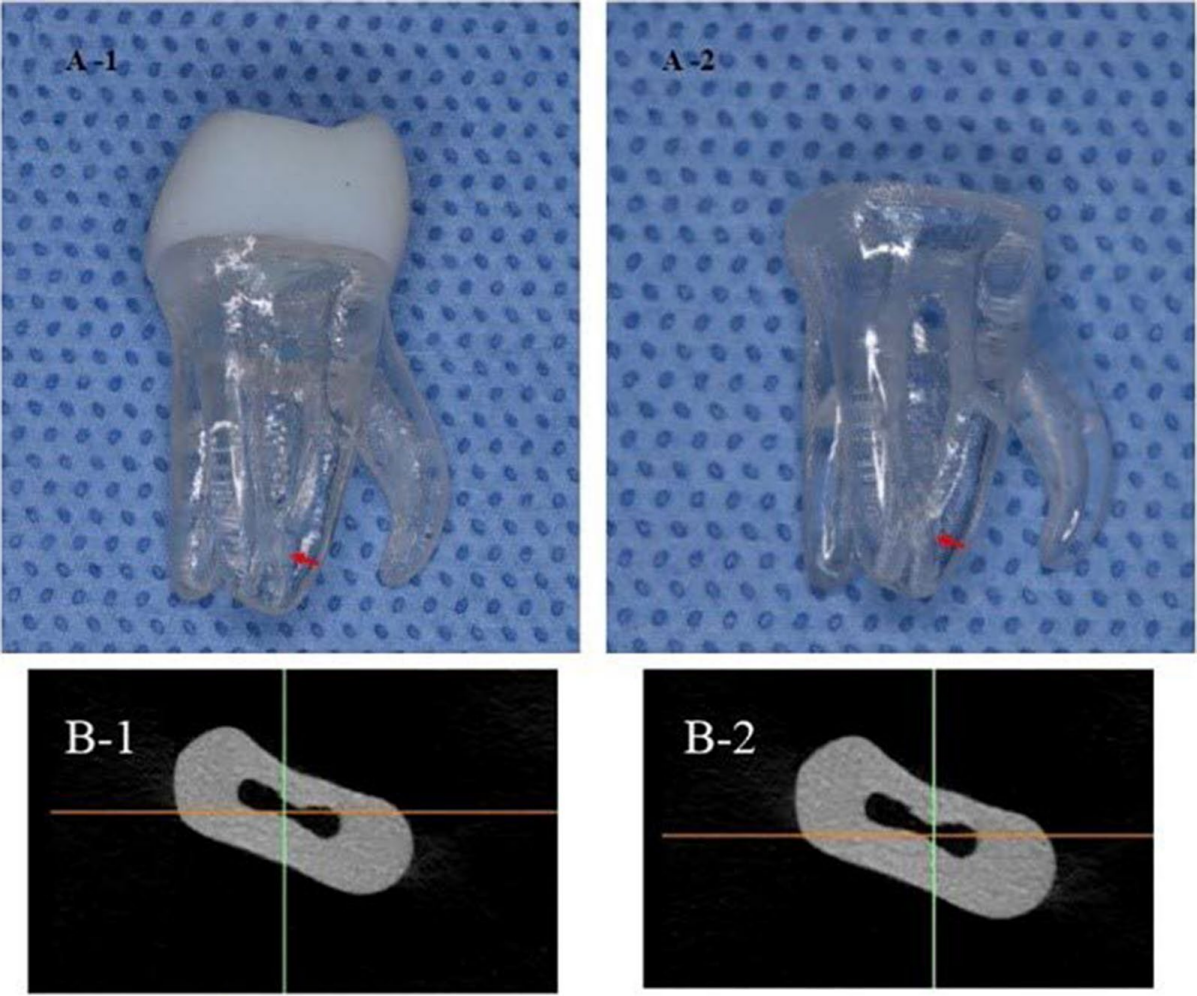
**A** 3D printed artificial teeth. Red arrows indicate isthmus.(1) Integral artificial molar teeth. (2) artificial molar teeth without crowns used in this experiment; **B** Superimposition images of the canals after preparation. From axis view, the differences between specimens could be negligible. (1) The overlapping image of sample 1 and 2. (2) The overlapping image of sample 1 and 3

After root canal preparation, artificial tooth specimens (n = 3) were scanned in order to evaluate the shape of the prepared canal space and discrepancies among specimens (Fig. 1). The differences between specimens was negligible, with similar volumes of the prepared space of the “band-shape isthmus” in the three samples (1.01982 ± 0.06 mm^3^).

**Fig. 2:**
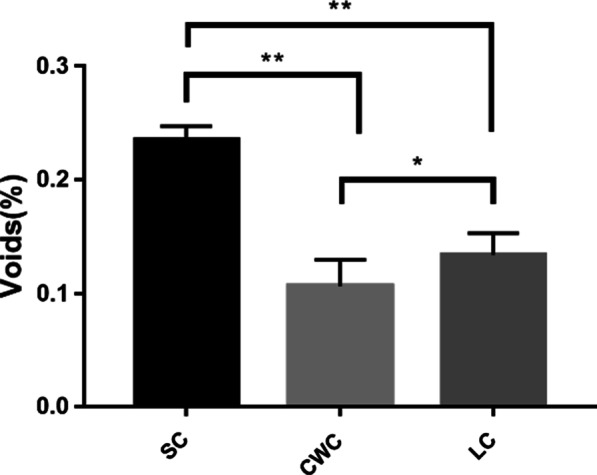
Volume percentage of voids after obturation in the isthmus areas in three groups. ‘*’ represents statistically significant differences between the groups (*p* < 0.05), while ‘* *’ represents *p* < 0.01.SC,single-cone technique; LC, lateral condensation technique; CWC,continuous wave of condensation technique

Analysis of micro-CT 3D reconstructions revealed the mean volume of voids in the focused area after obturation in the SC, CWC, and LC groups was 0.23436, 0.10668, and 0.13394 mm^3^ respectively, with percentage values of 22.98 % ± 1.19 %, 10.46 % ± 2.28 %, and 13.14 % ± 1.85 % (Fig. 2). Further, the filling percentages of the three groups were 77.02 %, 89.54 and 86.9 % correspondingly. The SC group was found to have a significantly greater volume percentage of voids in isthmus obturation (*P* < 0.01) than the other two techniques. In addition, the filled volume ratio in LC isthmus areas was significantly lower than that in the CWC group (*P* < 0.05).

**Fig. 3:**
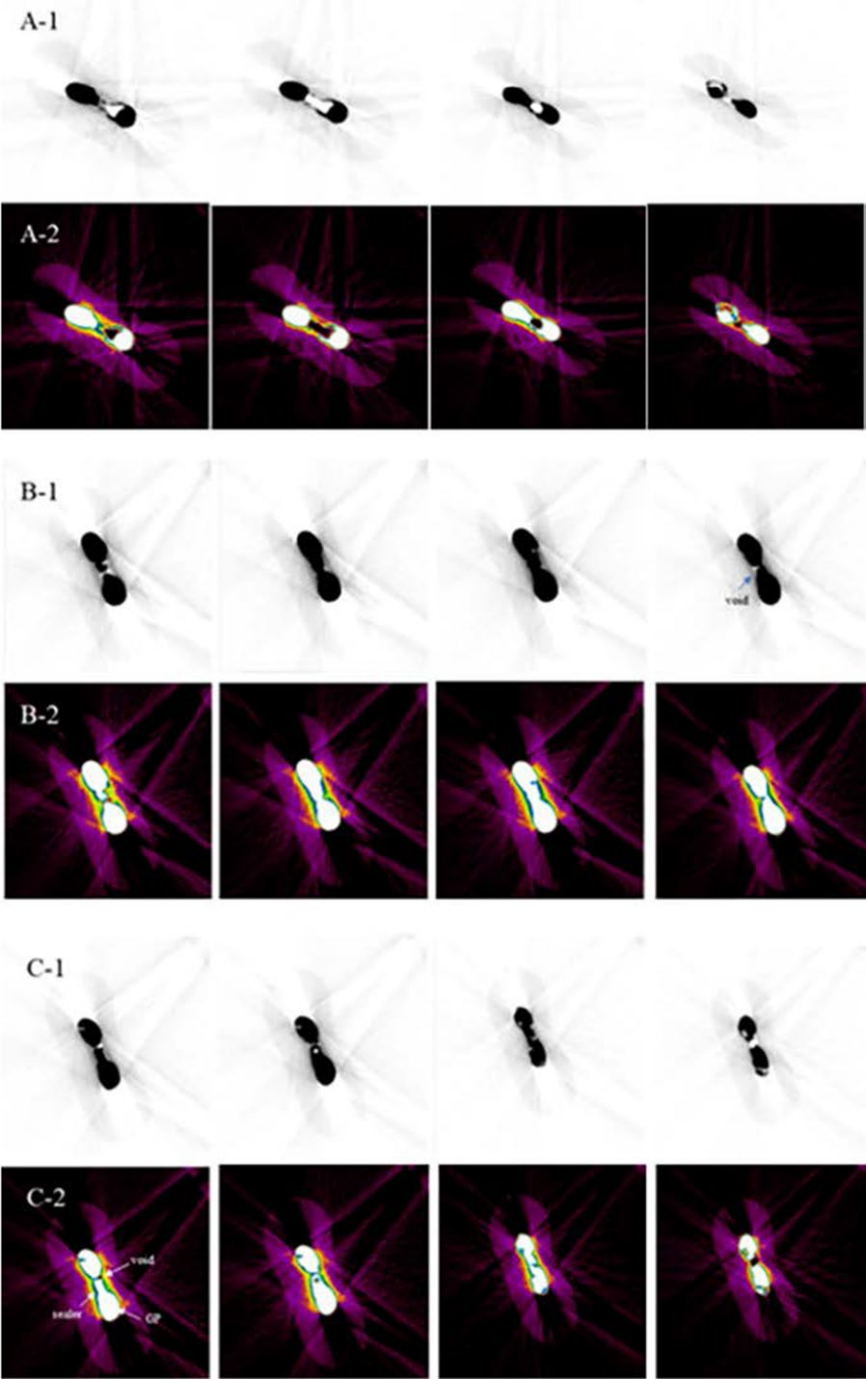
Representative micro-CT images of three specimens from three groups: **A** SC, **B** CWC and **C** LC technique groups. The lower row in (**A**), (**B**) and (**C**) is color-coded to distinguish the filling materials. The sealer, void, and gutta-percha (GP) are marked with arrows. (1) and (2) also present representative and consecutive micro-CT cross-sections of one specimen

**Fig. 4:**
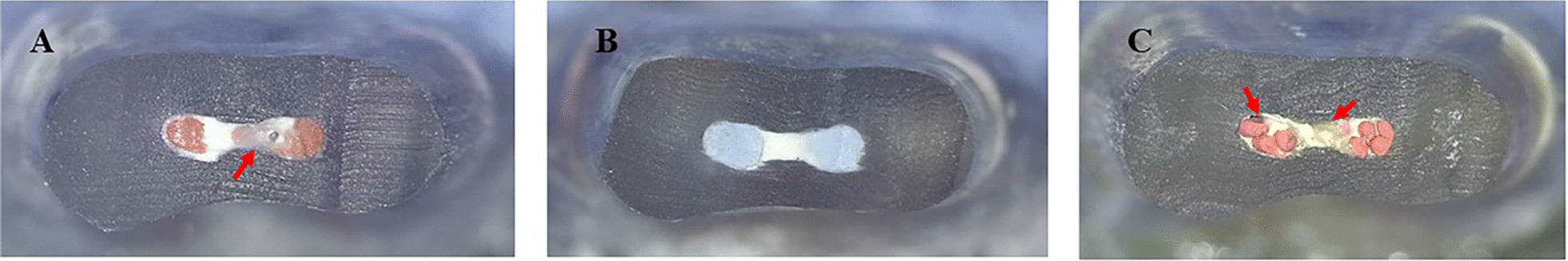
Operating microscope images (16x) of filling materials in the middle of isthmus in three groups: (**A**) SC group; (**B**) CWC group;(**C**) LC group.The arrow indicates a visible void

Two-dimensional axial cross-sections of representative specimens showed the voids in the root canal filling mass (Fig. 3). Voids were present both inside fillings as well as between the filling materials and root canal walls. Operating microscopic observation demonstrated that the CWC and LC groups were both well-packed, without voids in the middle of isthmuses. In the SC group, the void formation in the middle of the isthmus was clearly visible (Fig. 4).

## Discussion

Successful root canal therapy depends mainly on controlling pulp space infection, which can be achieved by good cleaning and shaping followed by three-dimensional filling of the entire root canal system [1–3, 6]. However, these steps are frequently challenged by the characteristics of root canal anatomy such as isthmuses. It is reported that the apical 5 mm region of the mandibular first molar mesial root canals tends to have the highest prevalence of isthmuses, which appear long oval in cross-sectional shape with the largest diameter in the buccolingual direction at 1 to 5 mm, which is not evident on periapical radiographs [4, 9–11]. Several studies have pointed out that increased risk of failure of endodontic treatment is associated with the presence of isthmus-containing canals, owing to the fact that this irregular area may harbor a significant number of microorganisms that might lead to treatment failure [22–24]. In the present study, the null hypothesis was rejected since significant differences were found among the obturation techniques.

Consequently, the majority of previous similar studies have used extracted natural molar teeth to reproduce the clinical situation of isthmus filling [9, 15, 18]. However, the wide variability of isthmuses makes standardization between groups difficult [10, 18]. Instead, custom-made 3D-printed artificial molar teeth, which had a fused mesial root with canals and a band-shaped isthmus in the apical 5 mm, were used in the present study.To the best of our knowledge, this is the first report to use these artificial teeth in a band-shaped isthmus study.The artificial teeth selected have the same type, volume, length and dimensions, guaranteeing the homogeneity of experimental groups, which makes it possible to obtain more reliable results in terms of the void percentages in the isthmus regions after obturation.

Although uniform samples were used in the experiment, the prepared canal shapes may have an influence on the obturation results. Therefore, in order to assess whether the shape of the isthmuses in the mesial root after preparation were consistent, three prepared artificial teeth were randomly selected to obtain micro-CT images as previously reported [21, 25]. The acquired images of the three samples were superimposed, and the results showed no obvious displacement in the location of the isthmus’ roof and floor of specimens. Thus the tooth-to-tooth variation can be considered negligible. This may probably because the original canal and isthmus space were quite wide, thus, the conventional root canal preparation had a limited impact on the root canal space of 3D-printed artificial teeth. Consequently, we could maintain a consistent isthmus space volume in all specimens.

Voids percentage has been used as a method to evaluate the quality of root canal filling [14–20], because bacteria and their byproducts may remain at a site in non-obturated areas and affect endodontic treatment outcome. Therefore, adequate sealing of the root canal system is required to prevent the spread of microorganisms and toxins [26].

Previous studies have reported slightly different criteria for dividing the root area to calculate the void percentage. Iglecias et al. [15] divided the root into three areas: apical, middle, and cervical, to assess the void percentage. Kim et al. [25] divided the apical region into 1–5 mm and 5–9 mm areas for measurement, because they considered this measurement to be more clinically relevant in root canal treatment success than measurements of the full canal length. Somma et al. [26]analyzed the entire root canal system as a whole without division into thirds. In the present study, a similar approach [26]was preferred and a statistically significant difference was detected between different obturation techniques. It is worth noting that according to the findings of the present study, all three obturation techniques were subject to failure, i.e., none were able to completely fill the isthmus region, which is similar to the findings of most previous studies [7, 14–20].

In this study, there were significantly more voids in the samples treated by the SC technique than with the CWC and LC methods in filling of isthmuses, with a percentage of 22.98 %. This result was consistent with that of several earlier studies [7, 17]. This may be because, unlike the other two techniques, the SC technique lacks vertical and lateral pressure during the obturation procedure. It just barely allows the use of a GP cone tapered according to the final shape of the canal, working as a key-and-lock system. Without pressure, it is difficult for the filling materials to enter the isthmus regions, therefore, there was a possibility of more and larger voids forming in the isthmus in the SC technique group. This may be the main reason why more obvious voids can be observed under the operating microscope compared to the CWC and LC groups. In this study, the middle of the isthmus, which was 3 mm away from the apex,were horizontally crossed to observe voids. Through the microscope observation,it is obviously to find that among three obturation techniques, the middle part of the isthmus was mostly filled with root canal sealer even in CWC group, which indicated that the filling technique based on sealer was worthy of attention.

However, other authors reached different conclusions in their studies compared with this research. Iglecias et al. [15]measured the volume of voids in the mesial root of the human mandibular molar in the apical one-third showing Vertucci Type II configuration canals, and found no significant difference between the SC and CWC groups in the apical area. In addition, a similar study conducted by Keles et al. [18] used micro-CT to measure the volume of voids in the band-shaped isthmuses in the mesial root of human mandibular first molars filled with AH plus sealer, and found similarities between the SC and CWC groups. Somma et al. [26] assessed straight canals, and they found no differences in root canal obturation or void distribution when comparing these two techniques. Voids and gaps within root canal fillings can be influenced by several factors [18], such as the experience of the clinician, the root canal mechanical preparation technique, differences in irrigation methods, the selected filling technique, the physical properties of the selected sealer, the focused anatomy configuration of the root canal system, the use of different scanning devices, diverse calculation softwares and so on. We speculated that these factors may be reasons for the differences between other studies and the outcomes of this research.

In addition, it has been reported that the SC technique provides inadequate obturation in oval root canals [7, 27, 28]. A micro-CT analysis of band-shaped isthmuses revealed that both the roof and floor of this anatomy exhibited a long, oval cross-sectional shape [10], thus lacking a full adaptation to the oval canal walls would complicate the obturation of isthmuses using the SC technique. The CWC technique has been reported to provide a more homogenous filling with significantly fewer voids when compared to either the SC or the LC technique [7, 18, 19, 21, 26, 28]. Homogeneous thermoplasticized GP with a certain liquidity, suitable vertical pressure, and tightly condensed backfilling make it more effective in filling complex root canal system [26–29]. In the present study, significant differences in the voids and more complete filling quality were found in the band-shaped isthmus region when comparing CWC with the cold-filling techniques. The better sealing,the better outcome for root canal therapy. Therefore, CWC technique may lead to a better prognosis for the tooth with band-shaped isthmus, which needed additional clinical studies to investigate.

However, Li et al. [30]demonstrated that the obturation quality, long-term outcome, and postoperative pain prevalence were similar between warm GP and cold LC obturation through a systematic review and meta-analysis. In addition, a retrospective analysis [31] reported that the overall success rate of nonsurgical endodontic treatment using EndoSequence Bioceramic Sealer and a single cone obturation technique was 90.9 %, which was higher than the reported success rate of initial root canal treatment (89.1 %) and retreatment (85.6 %) in a study with a large sample size [32]. Moreover, in a recent non-randomized clinical trial [33],the author used CBCT images and PA radiographs as loose criteria to compare the success rate of root canal treatments undertaken using a single cone technique and a calcium silicate sealer (BioRoot™ RCS) with a warm vertical condensation technique and AH plus, and the results showed no difference between those two groups. These studies showed that although the CWC technique achieved a lower void percentage in isthmus areas in the present study, cold filling techniques still resulted in a good clinical outcome in some aspects, especially the SC technique. Thus, further research is needed.

There were several limitations to this study. The 3D-printed artificial teeth lacked the microscopic structures characteristic of the dentin of natural teeth such as dentinal tubules, therefore, the adhesion between the endodontic filling material and the root canal wall could not be completely reproduced. Another consideration was that heating could alter the properties of the sealer, thus impacting on results of obturation. In addition, the outcome of this study mainly based on one band-shaped isthmus type, but the different isthmus types such as V-shaped or other irregular configurations still remained important factors for the void distribution produced by root canal filling techniques.Therefore,further investigations on different isthmus types should be conducted to get more information on obturation efficacy with different techniques for treatment of these anatomically challenging variations.

## Conclusions

Within the limitations of this in vitro study, our findings suggest that none of the root canal filling techniques produce void-free fillings in band-shaped isthmus obturation. The CWC yields superior obturation quality to that of SC and LC techniques in band-shaped isthmus area.

## Data Availability

All data involved in this study are available from corresponding author upon reasonable request.

## Abbreviations

micro-CT: micro-computed tomography
SC: single-cone technique
CWC: continuous wave of condensation technique
LC: lateral condensation technique
GP: gutta-percha
3D: three dimensions
MB: mesial-buccal canal
ML: mesial-lingual canal
WL: working length
